# Delirium as a predictor of mortality and disability among hospitalized patients in Zambia

**DOI:** 10.1371/journal.pone.0246330

**Published:** 2021-02-11

**Authors:** Justin K. Banerdt, Kondwelani Mateyo, Li Wang, Christopher J. Lindsell, Elisabeth D. Riviello, Deanna Saylor, Douglas C. Heimburger, E. Wesley Ely

**Affiliations:** 1 Department of Internal Medicine, Yale University School of Medicine, New Haven, Connecticut, United States of America; 2 Vanderbilt University School of Medicine, Nashville, Tennessee, United States of America; 3 Critical Illness, Brain Dysfunction, and Survivorship (CIBS) Center, Vanderbilt University Medical Center, Nashville, Tennessee, United States of America; 4 University of Zambia School of Medicine, Lusaka, Zambia; 5 University Teaching Hospital, Lusaka, Zambia; 6 Department of Biostatistics, Vanderbilt University Medical Center, Nashville, Tennessee, United States of America; 7 Beth Israel Deaconess Medical Center, Boston, Massachusetts, United States of America; 8 Harvard Medical School, Boston, Massachusetts, United States of America; 9 Johns Hopkins University School of Medicine, Baltimore, Maryland, United States of America; 10 Department of Medicine, Vanderbilt University Medical Center, Nashville, Tennessee, United States of America; 11 Vanderbilt Institute for Global Health, Nashville, Tennessee, United States of America; 12 Tennessee Valley Veteran’s Affairs Geriatric Research Education Clinical Center (GRECC), Nashville, Tennessee, United States of America; Clinca Geriatrica, ITALY

## Abstract

**Objective:**

To study the epidemiology and outcomes of delirium among hospitalized patients in Zambia.

**Methods:**

We conducted a prospective cohort study at the University Teaching Hospital in Lusaka, Zambia, from October 2017 to April 2018. The primary exposure was delirium duration over the initial 3 days of hospitalization, assessed daily using the Brief Confusion Assessment Method. The primary outcome was 6-month mortality. Secondary outcomes included 6-month disability, evaluated using the World Health Organization Disability Assessment Schedule 2.0.

**Findings:**

711 adults were included (median age, 39 years; 461 men; 459 medical, 252 surgical; 323 with HIV). Delirium prevalence was 48.5% (95% CI, 44.8%-52.3%). 6-month mortality was higher for delirious participants (44.6% [39.3%-50.1%]) versus non-delirious participants (20.0% [15.4%-25.2%]; *P* < .001). After adjusting for covariates, delirium duration independently predicted 6-month mortality and disability with a significant dose-response association between number of days with delirium and odds of worse clinical outcome. Compared to no delirium, presence of 1, 2 or 3 days of delirium resulted in odds ratios for 6-month mortality of 1.43 (95% CI, 0.73–2.80), 2.20 (1.07–4.51), and 3.92 (2.24–6.87), respectively (*P* < .001). Odds of 6-month disability were 1.20 (0.70–2.05), 1.73 (0.95–3.17), and 2.80 (1.78–4.43), respectively (*P* < .001).

**Conclusion:**

Among hospitalized medical and surgical patients in Zambia, delirium prevalence was high and delirium duration independently predicted mortality and disability at 6 months. This work lays the foundation for prevention, detection, and management of delirium in low-income countries. Long-term follow up of outcomes of critical illness in resource-limited settings appears feasible using the WHO Disability Assessment Schedule.

## Introduction

Delirium is a form of acute neurologic injury that is common among critically ill patients [1]. Studies from high-income countries have shown that delirium is a strong, independent predictor of long-term mortality [2–5], as well as prolonged cognitive and functional impairment [6–12]. Although delirium is now considered an issue of public health importance among critically ill patients in high-income countries, where widespread delirium prevention and management efforts are now underway across intensive care settings [13, 14], limited information exists about the epidemiology and outcomes of delirium in low- and middle-income countries (LMICs).

A recent review [15] of the delirium literature from sub-Saharan Africa found that most studies contained significant methodological concerns, and demonstrated widely varying point estimates of delirium prevalence. Prior studies have generally not used validated criteria for delirium assessment, have focused on psychiatric settings, and have not rigorously examined the relationship of delirium to outcomes [15]. One prospective study [16] of 160 mechanically ventilated patients in four intensive care units (ICUs) in Kampala, Uganda found a 51% prevalence of delirium; it was not powered to detect an association of delirium with mortality and only included in-hospital outcomes.

Furthermore, little is known about the long-term outcomes of critical illness in resource-limited settings, though the global burden of critical illness is disproportionately high in LMICs [17, 18]. A recent review [19] found no data on long-term functional outcomes of critical illness in resource-limited settings and only one study of post-ICU mortality [20]. Although progress has been made on the development and validation of mortality prediction scores in sub-Saharan Africa, these have been limited to short-term in-hospital mortality prediction [21, 22]. In addition, recent studies of fluid resuscitation in sepsis [23, 24] have demonstrated that results from studies of critically ill patients in high-income settings cannot be reliably extrapolated to LMICs, underlining the need for long-term critical illness outcomes research in resource-limited settings.

We designed this prospective cohort investigation to study the epidemiology and outcomes of delirium among adult medical and surgical patients admitted to a national referral hospital in Zambia. We sought to test the hypothesis that duration of delirium in LMIC patients is an independent predictor of 6-month mortality and disability and that long-term follow up of functional outcomes of critical illness is feasible in a resource-limited setting.

## Methods

### Study design and population

We conducted a prospective cohort study at the University Teaching Hospital (UTH), a 1655-bed national referral hospital in Lusaka, Zambia with approximately 17,500 acute admissions annually. UTH is the only tertiary referral hospital for Zambia [25], an LMIC with a population of approximately 18 million. While the hospital had only 10 ICU beds at the time of the study, previous research [25] identified that approximately 45% of medical and surgical patients admitted to non-ICU units at UTH met criteria for critical care triage. The University of Zambia Biomedical Research Ethics Committee, the Zambia National Health Research Authority, and the Vanderbilt University Institutional Review Board (IRB) granted ethical approval. Written informed consent was obtained from patients or their legally authorized representatives prior to enrollment in the study.

Enrollment occurred from October 30, 2017, to April 5, 2018. Patients aged 18 years or older were eligible. The only exclusion criterion was inability to understand one of the three primary local languages (English, Nyanja, and Bemba). Each weekday morning, we screened all new medical and surgical admissions, including overnight admissions, from the past 24 hours for inclusion in the study. Enrollment occurred in the medical and surgical admission wards. Due to high patient volumes, a convenience sample approach was taken for patient enrollment such that enrollment was stopped once the maximum number of patients that could be feasibly enrolled each day was reached (about 10 patients daily, or approximately 20% of total daily admissions). To address potential lack of representativeness in the convenience sample, a separate study was conducted in the same setting in which all patients admitted over seven days were enrolled from April 30 through May 6, 2018 to examine delirium point prevalence.

### Exposures

Delirium assessment was conducted using the modified Brief Confusion Assessment Method (bCAM) [26, 27], a validated instrument for use by physicians and non-physicians to assess delirium in acutely ill adults. Participants were considered delirious if they demonstrated an acute change in or fluctuation in the course of their mental status, inattention and either disorganized thinking or an altered level of consciousness. Standard guidelines were followed to create English, Nyanja, and Bemba language versions of the bCAM through translation and back-translation of the instrument to verify the accuracy of the translation. Based on feedback from local clinicians, two minor cultural adaptations to the instrument were made to ensure that it was not biased against illiterate patients or those with limited formal education: the test for inattention in feature 2 was changed from letters to a list of single digit numbers in which the patient was asked to squeeze the examiner’s hand whenever the number “7” was called out; in feature 4 we used bags of sugar instead of pounds for the weight comparison question (a more widely understood measure in this population) and removed reference to “fish in the sea” in another question (due to Zambia being land-locked).

Study nurses who were fluent in the local languages were trained by the study investigators in the use of the bCAM. A senior physician at UTH not associated with the study then evaluated the nurses for correct use of the bCAM across all language versions. Study nurses assessed patients for delirium upon enrollment and again at 24 hours and 48 hours (i.e., once daily) post-enrollment unless the patient died or was discharged. Patients therefore received up to three delirium evaluations over their first 3 days of hospitalization. For the 7-day point prevalence study, patients were only evaluated once for delirium on admission.

### Outcomes

The primary outcome was 6-month mortality. Secondary outcomes included 28-day mortality and 6-month disability among survivors. Mortality and date of death were assessed either by consulting in-hospital death records or by contacting next-of-kin by mobile phone. Blinded study personnel called patients or next-of-kin at 28 days and 6 months after enrollment to ascertain vital status and disability. Disability at 6 months was measured using the World Health Organization Disability Assessment Schedule 2.0 (WHODAS 2.0) [28]. WHODAS 2.0 is an instrument developed by the WHO to provide a standardized method for assessing disability across cultures; it has been validated for use in LMICs [29]. The instrument consists of 36 items that assess the degree of difficulty the respondent has in carrying out different tasks using a 5-point Likert scale. These items are grouped into six domains of functioning: cognition (understanding and communicating), mobility (moving and getting around), self-care (hygiene, dressing, eating and staying alone), getting along (interacting with other people), life activities (domestic responsibilities, leisure, work and school), and participation (joining in community activities). The six domain-specific scores are averaged to yield a total disability score ranging from 0 to 100 with higher scores indicating greater levels of disability. The 36-item interviewer-administered version of the WHODAS 2.0, validated for administration over the phone [30], translated into the local languages and back-translated to ensure accuracy, was used in this investigation.

REDCap was used for secure entry, storage, and management of all study data [31].

### Statistical analysis

Analysis of 6-month mortality, 6-month disability, and 28-day mortality only included participants who survived to 3 days, so that the effect of cumulative days of delirium exposure over the initial 3 days of hospitalization could be modeled for outcomes. We performed single imputation for missing delirium assessments based on the delirium status on the prior day and the day after. For the 34 participants who were comatose at one or more timepoints, their delirious status at these time points was changed from unassessable to positive for outcomes analysis. Duration of delirium was calculated from the daily bCAM and ranged from 0 to 3 days. Pearson’s chi-square test was used for comparing delirium prevalence and unadjusted mortality between subgroups.

For regression modeling of 6-month mortality, 6-month disability, and 28-day mortality, we adjusted for seven *a priori* defined covariates: age, sex, education, income, Universal Vital Assessment (UVA) score [21], HIV status, and current antituberculosis treatment. The UVA severity of illness (SOI) score has been validated in 6 sub-Saharan African countries among 5,573 adult patients including those with HIV and sepsis. We also collected data *a priori* on all patients using the Modified Early Warning Score (MEWS) [32, 33] and the quick Sequential Organ Failure Assessment (qSOFA) [34–36] SOI scores. Prior to analysis we chose to use the UVA score as our primary SOI score because it was validated in sub-Saharan Africa. However, we ran models that included all three scores (UVA, MEWS, and qSOFA) and found similar results. In order to keep HIV as a separate variable in the regression modeling, we modified the UVA score by removing HIV from the score calculation; doing so has been shown to have minimal impact on the score’s performance [21].

We analyzed 6-month mortality using a binary logistic regression model rather than survival analysis due to difficulty in ascertaining exact dates of death between 1 and 6 months due to recall bias. 28-day mortality was analyzed using a Cox Proportional Hazard regression model and Kaplan-Meier survival analysis. For 6-month disability we fitted a proportional odds logistic model. To incorporate death as an ordinal outcome in this model using the WHODAS disability scale (which ranges from 0–100), we assigned an outcome score of 101 for participants who died prior to 6-month assessment. Tests for linear trend were conducted to assess for association between delirium dose as an ordinal variable and all three outcomes.

Analyses were performed using R version 3.5.2.

## Results

**Fig 1:**
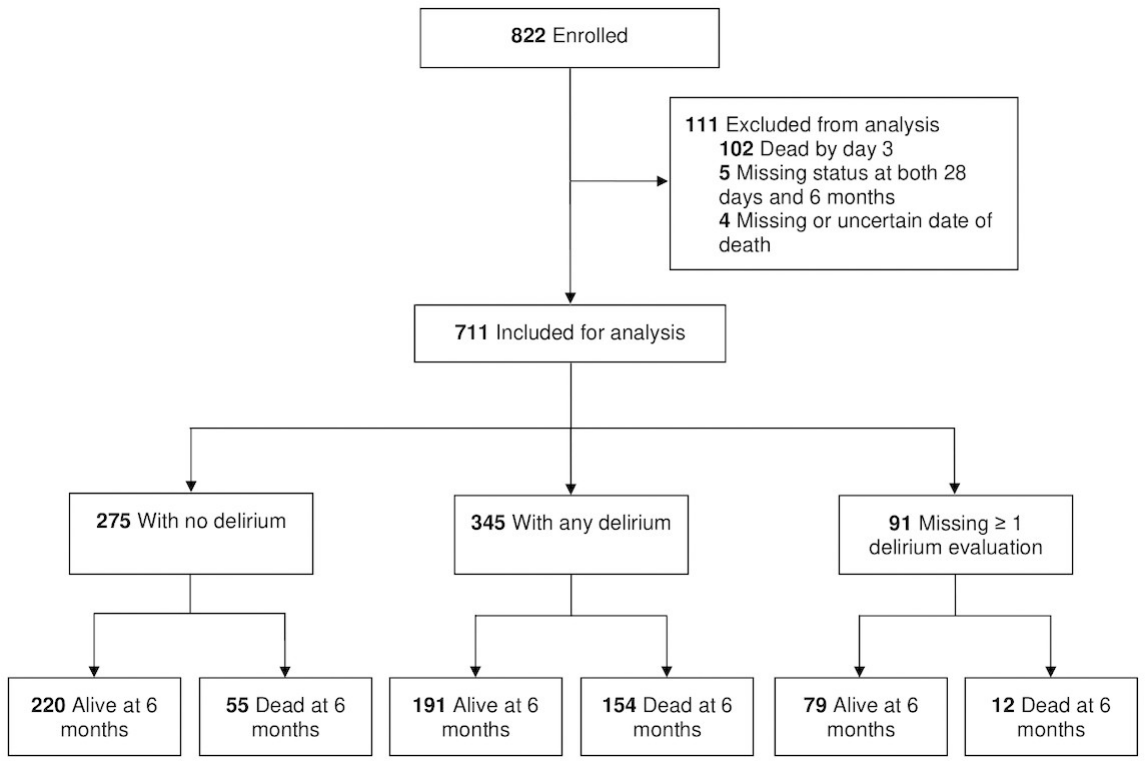
Participant enrollment, inclusion in analysis, and follow-up. This figure displays the number of participants enrolled, included for analysis, and followed up at 6 months by delirium status and vital status. No delirium and any delirium were defined as bCAM negative on all 3 evaluations and at least one positive bCAM evaluation, respectively. Missing ≥ 1 delirium evaluation was defined as a participant missing at least one bCAM evaluation with all others negative. Missing delirium evaluations were imputed in the multivariable outcomes analysis. Of 345 participants with any delirium, 154 died by 6-month follow-up.

A total of 822 patients met eligibility criteria and were enrolled into the study. Of these patients, 102 who died on or before day 3 of hospitalization were excluded from the primary analysis and 9 were excluded due to either missing vital status or uncertain date of death (overall loss to follow-up at 6 months: 1.3%) (Fig 1). The remaining 711 patients (459 medical and 252 surgical) were included in the primary analysis (Table 1). Additionally, 330 patients were enrolled separately in the 7-day point prevalence study.

**Table 1:** Baseline characteristics of participants by delirium exposure^a^.

	No Delirium (N = 275)	Any Delirium (N = 345)	Missing (N = 91)
Age, mean (SD), y	40 (15)	45 (17)	39 (15)
Male sex, No. (%)	187 (68.0)	216 (63.0)	58 (64.0)
Admission type, No. (%)			
Medical admission	161 (58.0)	239 (69.0)	59 (65.0)
Surgical admission	114 (42.0)	106 (31.0)	32 (35.0)
Level of education, No. (%)			
Never attended	7 (2.6)	18 (5.3)	3 (3.4)
Grade 1–7	86 (31.9)	121 (35.7)	21 (23.6)
Grade 8–12	143 (53.0)	156 (46.0)	50 (56.2)
University	34 (12.6)	44 (13.0)	15 (16.9)
Average monthly income, No. (%)^b^			
0–500 Kwacha (0–50 USD)	127 (46.4)	185 (54.1)	37 (41.1)
501–1500 Kwacha (50.1–150 USD)	66 (24.1)	80 (23.4)	21 (23.3)
>1500 Kwacha (>150 USD)	81 (29.6)	77 (22.5)	32 (35.6)
UVA Score, median (IQR)^c^	2.0 (0.75–4.0)	6.0 (4.0–8.0)	2.0 (0.0–4.0)
Modified UVA Score, median (IQR)^d^	1.5 (0.0–3.0)	5.0 (4.0–7.0)	2.0 (0.0–2.0)
MEWS Score, median (IQR)^e^	3.0 (2.0–4.0)	4.0 (3.0–5.0)	2.0 (1.0–4.0)
qSOFA Score ≥2, No. (%)^f^	61 (22.3)	181 (53.2)	14 (15.7)
Glasgow Coma Scale Score <15, No. (%)^g^	12 (4.4)	269 (78.2)	7 (7.8)
Upper arm circumference, median (IQR)^h^	26.6 (23.0–30.0)	26.2 (22.8–31.0)	28.0 (23.8–32.0)
Living with HIV, No. (%)	126 (45.8)	159 (46.4)	38 (42.7)
Time since diagnosis, median (SD), d	730 (91–2922)	365 (21–1461)	1644 (639–3379)
On ARV therapy, No. (%)	104 (83.9)	117 (75.0)	35 (94.6)
ARV therapy duration, median (IQR), d	1096 (167–3105)	730 (61–1826)	1826 (730–3470)
History of tuberculosis infection, No. (%)^i^	80 (29.5)	91 (27.2)	16 (18.0)
On antituberculosis treatment	32 (41.0)	44 (48.9)	6 (37.5)
Admission diagnosis, No. (%)^j^			
Tuberculosis	63 (23.0)	94 (27.0)	10 (11.0)
Anemia	54 (20.0)	75 (22.0)	13 (14.0)
Pneumonia	37 (13.5)	52 (15.1)	8 (8.8)
Renal dysfunction	32 (11.6)	55 (15.9)	7 (7.7)
Trauma, burns, penetrating injury	35 (12.7)	25 (7.2)	16 (17.6)
Sepsis	20 (7.3)	50 (14.5)	4 (4.4)
Head/spinal injury	13 (4.7)	59 (17.1)	2 (2.2)
Meningitis/encephalitis	14 (5.1)	51 (14.8)	5 (5.5)
Hypertension/hypertensive urgency	14 (5.1)	43 (12.5)	8 (8.8)
Shock	23 (8.4)	32 (9.3)	3 (3.3)
bCAM positive at enrollment, No. (%)	0 (0.0)	300 (87.0)	0 (0.0)
Medical treatments ordered on admission^k^			
IVFs, mean (SD), liters	1.14 (1.51)	1.26 (1.54)	0.86 (1.36)
Antibiotics, No. (%)	160 (58.0)	218 (63.0)	38 (42.0)
Sedatives, No. (%)	4 (1.5)	25 (7.3)	4 (4.4)
Anticonvulsants, No. (%)	1 (0.4)	7 (2.0)	0 (0.0)
Opioids, No. (%)	31 (11.3)	23 (6.7)	8 (8.9)
Vasopressors, No. (%)	7 (2.5)	11 (3.2)	0 (0.0)

Abbreviations: USD, United States Dollar; UVA, Universal Vital Assessment; qSOFA, quick Sequential Organ Failure Assessment; MEWS, Modified Early Warning Score; GCS, Glasgow Coma Scale; HIV, Human Immunodeficiency Virus; ARV, antiretroviral; TB, Mycobacterium tuberculosis; bCAM, brief Confusion Assessment Method; IVFs, Intravenous Fluids.

^a^No delirium is defined as all negative bCAM evaluations. Any delirium is defined as one or more positive bCAM evaluations. Missing is defined as one or more missing bCAM evaluations with all others negative. Missing delirium evaluations were imputed for the multivariable outcomes analysis.

^b^Average monthly income was recorded in local currency (Kwacha) and then converted to US dollar equivalent based on an exchange rate of 10 Kwacha to 1 US dollar.

^c^Scores on the UVA range from 0–13, with higher scores indicating greater risk of in-hospital mortality. A UVA score greater than 4 indicates patients at high risk for in-hospital mortality.

^d^The UVA was modified by removing HIV from the score. The modified UVA ranges from 0–11, with higher scores indicating greater risk of in-hospital mortality. The modified UVA without HIV was used in the primary multivariable regression models for delirium duration and outcomes.

^e^Scores on the MEWS range from 0–14, with higher scores (≥5) indicating greater risk of ICU admission or death within 60 days.

^f^Scores on the qSOFA range from 0–3, with scores ≥2 indicating high risk for in-hospital mortality.

^g^Scores on the Glasgow Coma Scale range from 3–15, with lower scores indicating lower level of consciousness.

^h^Upper arm circumference is used as a measure of nutrition, with values ≤24.0 cm in adults indicating low BMI and risk for malnutrition.

^i^History of tuberculosis infection includes smear positive and smear negative cases.

^j^Only the 10 most common admission diagnoses are included (26 total were coded), so percentages may not sum to 100. Admission diagnoses are listed in order of prevalence, from most prevalent to less prevalent. Some patients received multiple diagnoses.

^k^Medical treatments ordered on admission only include those ordered in the patient chart and does not specify whether patients received the treatment.

### Delirium prevalence and baseline characteristics

Delirium prevalence (positive bCAM) at enrollment was 47.0% (95% CI, 43.5%-50.5%) in the primary cohort versus 43.6% in the 7-day point prevalence study. Total delirium prevalence was 48.5% (95% CI, 44.8%-52.3%) over the 3-day observation period. Total delirium prevalence was slightly higher for persons with HIV (49.2% [95% CI, 43.7%-54.8%]) versus HIV-negative participants (45.8% [95% CI, 40.4%-51.3%]; *P* = .045), and higher for medical admissions (52.1% [95% CI, 47.4%-56.7%]) versus surgical admissions (42.1% [95% CI, 35.9%-48.4%]; *P* = .021).

Delirious participants had a median UVA severity of illness score of 6, indicating a population with a high risk of death. The prevalence of HIV in the cohort was 45.4%. Among those with HIV and delirium, the median length of time since HIV diagnosis was 365 days, and 75.0% were on antiretroviral treatment. Tuberculosis (TB) burden was substantial, with 27.2% of delirious participants having a history of TB, and of those, 48.9% were receiving antituberculosis treatment at the time of study enrollment.

### Primary outcome

**Fig 2:**
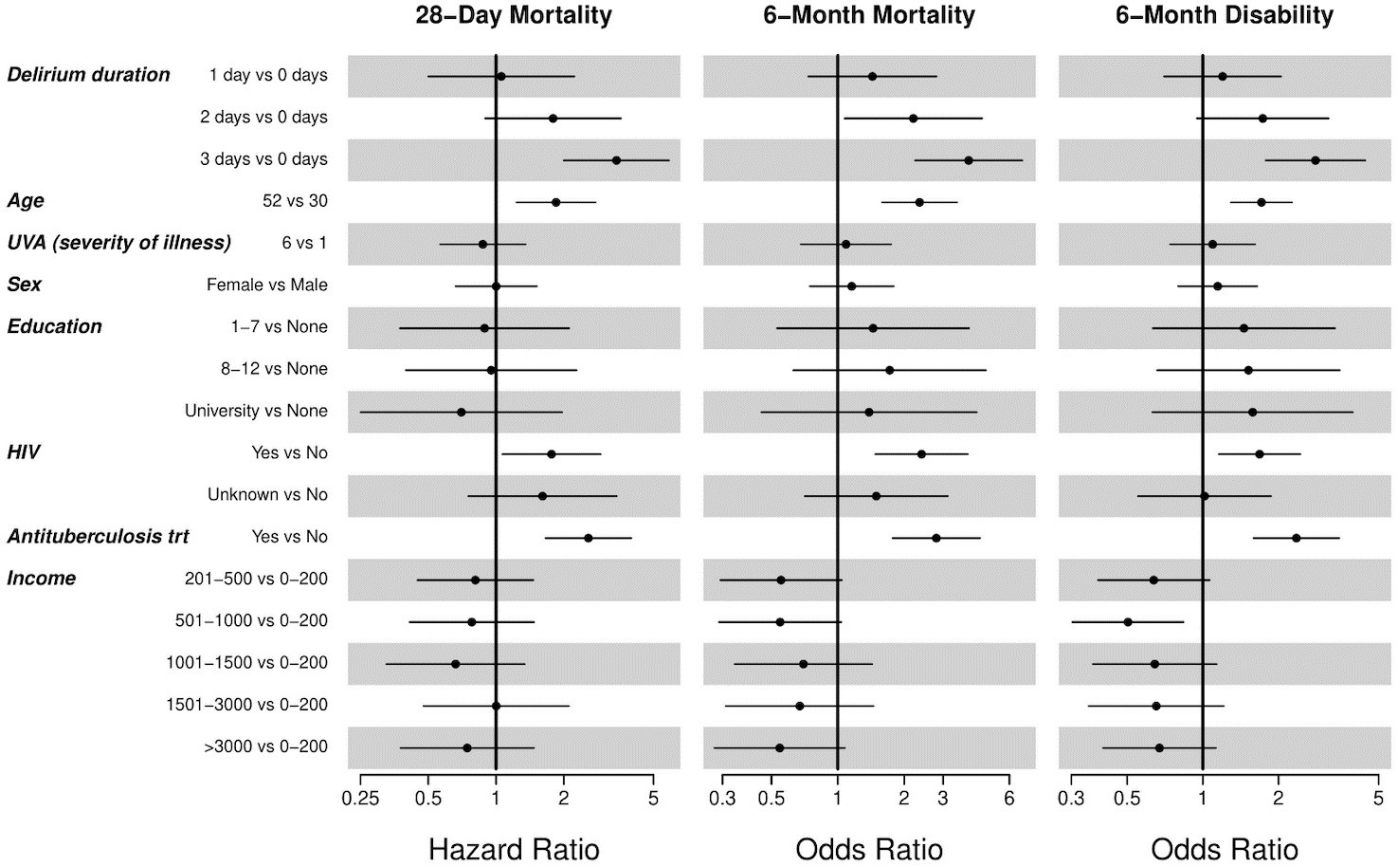
Forest plot of multivariable regression analysis of clinical outcomes. This figure displays the odds and hazard ratios (x-axis) for the covariates (y-axis) included in the multivariable regression analyses of clinical outcomes, which are displayed in separate columns for each outcome (28-day mortality, 6-month mortality, and 6-month disability). Odds and hazard ratio values are indicated by circles with horizontal lines indicating the associated 95% confidence intervals and vertical lines indicating an odds or hazard ratio of 1. The comparator and reference values for each covariate are listed following the covariate name on the y-axis. For age and UVA, the comparator values represent the 75^th^ and 25^th^ percentile. Duration of delirium was calculated from the daily bCAM and ranged from 0 to 3 days. In the regression model, outcomes for cumulative days of delirium (1, 2, or 3 days) are compared to outcomes for 0 days of delirium. After controlling for seven *a priori* defined covariates (age, UVA severity of illness score, sex, education, HIV status, antituberculosis treatment, and income), delirium duration was independently predictive of higher odds and hazard ratios for 28-day mortality, 6-month mortality, and 6-month disability (*P* < .001). For example, 3 days of delirium was predictive of higher odds for 6-month mortality and disability of 3.92 (95% CI, 2.24–6.87) and 2.80 (95% CI, 1.78–4.43), respectively (see Table 2).

Delirious participants had higher 6-month mortality, 44.6% (95% CI, 39.3%-50.1%) versus 20.0% (95% CI, 15.4%-25.2%) for non-delirious participants (*P* < .001). After controlling for seven *a priori* defined covariates, delirium duration was an independent predictor of greater 6-month mortality in multivariable analysis (Fig 2). Compared to no delirium, presence of 1, 2 or 3 days of delirium was predictive of higher odds of 6-month mortality of 1.43 (95% CI, 0.73–2.80), 2.20 (95% CI, 1.07–4.51), and 3.92 (95% CI, 2.24–6.87), respectively (*P* < .001 for delirium duration) (Table 2). A test for linear trend found a significant, dose-response association between increasing delirium exposure (i.e., higher delirium days) and greater odds of 6-month mortality (*P*<0.001). Although UVA was not predictive of 6-month mortality, all models were also run with MEWS, which was predictive of 6-month mortality (OR 1.95 [95% CI, 1.27–2.99]; P = .002), and qSOFA SOI scores as well and similar results were found with no qualitative differences (Table 3).

**Table 2:** Multivariable regression analysis of delirium duration and clinical outcomes^a^.

	28-Day Mortality			6-Month Mortality			6-Month Disability		
	HR	95% CI	*P*^c^	OR	95% CI	*P*	OR	95% CI	*P*
Delirium duration^b^			< .001			< .001			< .001
1 day vs 0 days	1.06	(0.50–2.23)		1.43	(0.73–2.80)		1.20	(0.70–2.05)	
2 days vs 0 days	1.79	(0.89–3.59)		2.20	(1.07–4.51)		1.73	(0.95–3.17)	
3 days vs 0 days	3.42	(2.00–5.87)		3.92	(2.24–6.87)		2.80	(1.78–4.43)	

^a^Seven *a priori* defined covariates were controlled for in the multivariable regression analysis of delirium duration and clinical outcomes, including age, sex, education, income, severity of illness (UVA score with HIV removed), HIV status, and current antituberculosis treatment.

^b^Duration of delirium was calculated from the daily bCAM and ranged from 0 to 3 days. In the regression model, outcomes for cumulative days of delirium (1, 2, or 3 days) are compared to outcomes for 0 days of delirium.

^c^*P* values are for cumulative days of delirium and are adjusted for seven *a priori* defined covariates.

**Table 3:** Multivariable regression analysis of clinical outcomes including UVA, MEWS, and qSOFA severity of illness scores^a^.

	28-Day Mortality			6-Month Mortality			6-Month Disability		
Covariate	HR	95% CI	*P*	OR	95% CI	*P*	OR	95% CI	*P*
Delirium duration^b^			< .001			< .001			< .001
1 vs 0 days	1.04	(0.49–2.19)		1.49	(0.77–2.87)		1.06	(0.65–1.73)	
2 vs 0 days	1.89	(0.94–3.81)		2.97	(1.42–6.24)		1.89	(1.03–3.48)	
3 vs 0 days	4.04	(2.31–7.08)		4.87	(2.70–8.79)		2.72	(1.72–4.30)	
Age^c^									
52 vs 30 years	1.80	(1.20–2.68)	.007	2.35	(1.59–3.49)	< .001	1.55	(1.20–2.01)	.002
UVA^d^									
6 vs 1	0.76	(0.48–1.20)	.238	0.69	(0.43–1.11)	0.125	0.83	(0.57–1.19)	.299
MEWS^e^									
5 vs 2	1.56	(1.06–2.32)	.025	1.95	(1.27–2.99)	.002	1.43	(1.04–1.98)	.03
qSOFA^f^									
2 vs 1	0.83	(0.58–1.19)	.322	1.03	(0.72–1.46)	.873	1.08	(0.83–1.41)	.561
Sex									
Female vs Male	1.01	(0.66–1.54)	.95	1.07	(0.69–1.68)	.757	1.08	(0.76–1.53)	.462
Education			.902			.730			.794
1–7 vs None	0.90	(0.38–2.16)		1.36	(0.49–3.76)		1.24	(0.56–2.76)	
8–12 vs None	0.96	(0.40–2.30)		1.59	(0.57–4.39)		1.21	(0.54–2.68)	
Univ. vs None	0.75	(0.27–2.12)		1.26	(0.40–3.95)		1.32	(0.55–3.19)	
HIV			.077			.002			.14
Yes vs No	1.75	(0.93–3.33)		2.94	(1.61–5.26)		1.61	(1.05–2.44)	
Unknown vs No	1.00	(0.43–2.33)		1.96	(0.81–4.76)		1.92	(0.97–3.85)	
TB treatment									
Yes vs No	2.88	(1.73–4.82)	< .001	2.49	(1.46–4.23)	.001	2.20	(1.39–3.46)	.002
Income^g^			.817			.302			.158
200–500 vs 0–200	0.83	(0.46–1.51)		0.55	(0.29–1.05)		0.71	(0.43–1.16)	
501–1000 vs 0–200	0.81	(0.42–1.54)		0.54	(0.28–1.03)		0.52	(0.32–0.85)	
1001–1500 vs 0–200	0.64	(0.31–1.30)		0.68	(0.33–1.42)		0.62	(0.36–1.06)	
1501–3000 vs 0–200	1.05	(0.50–2.22)		0.70	(0.32–1.52)		0.70	(0.39–1.27)	
>3000 vs 0–200	0.78	(0.39–1.56)		0.56	(0.28–1.12)		0.74	(0.45–1.21)	

Abbreviations: UVA, Universal Vital Assessment; MEWS, Modified Early Warning Score; qSOFA, quick Sequential Organ Failure Assessment; HIV, Human Immunodeficiency Virus.

^a^Nine *a priori* defined covariates were controlled for in this multivariable regression analysis of delirium duration and clinical outcomes, including age, sex, education, income, UVA score, MEWS score, qSOFA score, HIV status, and antituberculosis treatment. This is a secondary regression analysis including UVA, MEWS, and qSOFA severity of illness scores.

^b^Delirium duration was calculated from the daily bCAM and ranged from 0 to 3 days. In the regression model, outcomes for cumulative days of delirium (1, 2, or 3 days) are compared to outcomes for 0 days of delirium. *P* values for delirium duration are for cumulative days of delirium and are adjusted for nine *a priori* defined covariates, listed above.

^c^The comparator values for age in the model (52 years vs 30 years) represent the 75^th^ and 25^th^ percentile for age in the cohort, respectively.

^d^Scores on the UVA range from 0–13, with higher scores indicating greater risk of in-hospital mortality. A UVA score greater than 4 indicates patients at high risk for in-hospital mortality. The comparator values for UVA in the model (6 vs 1) represent the 75^th^ and 25^th^ percentile for UVA in the cohort, respectively.

^e^Scores on the MEWS range from 0–14, with higher scores (≥5) indicating greater risk of ICU admission or death within 60 days.

^f^Scores on the qSOFA range from 0–3, with scores ≥2 indicating patients at high risk for in-hospital mortality.

^g^Income levels are for average monthly income in local Zambian currency (Kwacha). The conversion for Kwacha to US dollars is approximately 10:1 (e.g., 0–200 Kwacha is equivalent to 0–20 US dollars).

### Secondary outcomes

**Fig 3:**
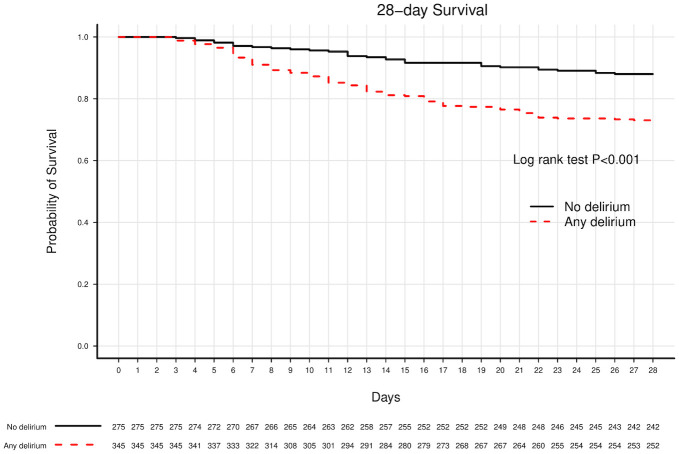
Kaplan-Meier analysis of delirium and 28-day survival. This figure displays the 28-day Kaplan-Meier survival curves for participants with any delirium (dashed red line) and no delirium (solid black line). Any delirium and no delirium were defined as at least one positive bCAM assessment and all negative bCAM assessments, respectively. Only participants who survived to 3 days and had no missing delirium evaluations were included in the analysis (N = 620). 28-day survival for participants with any delirium was 73.04% versus 88.0% for participants with no delirium. A log rank test showed that participants with any delirium had significantly lower unadjusted 28-day survival compared to participants with no delirium (*P* < .001).

Delirious participants had higher 28-day mortality, 27.0% (95% CI, 22.3%-32.0%) versus 12.0% (95% CI, 8.4%-16.4%) for non-delirious participants (*P* < .001) (Fig 3). After controlling for covariates, delirium duration was independently predictive of 28-day mortality in a dose-response relationship (Fig 2). Compared to no delirium, presence of 1, 2 or 3 days of delirium was predictive of hazard ratios for 28-day mortality of 1.06 (95% CI, 0.50–2.23), 1.79 (95% CI, 0.89–3.59), and 3.42 (95% CI, 2.00–5.87), respectively (*P* < .001 for delirium duration; test for linear trend *P*<0.001) (Table 2).

Delirium duration was an independent predictor of 6-month disability in a multivariable model adjusting for relevant covariates (Fig 2), with a significant dose-response relationship between days of delirium exposure and 6-month disability. Compared to no delirium, presence of 1, 2 or 3 days of delirium was predictive of odds for greater 6-month disability of 1.20 (95% CI, 0.70–2.05), 1.73 (95% CI, 0.95–3.17), and 2.80 (95% CI, 1.78–4.43), respectively (*P* < .001 for delirium duration; test for linear trend *P*<0.001) (Table 2).

## Discussion

This prospective cohort study found that delirium was significantly and independently associated with higher mortality and greater disability at 6 months among hospitalized medical and surgical patients at a national referral hospital in Zambia with a large burden of HIV, TB, and critical illness. Nearly 50% of patients in this population had delirium, a rate comparable to that of ICU patients in high-income countries [1]. Those with delirium had a 44.6% 6-month mortality rate, which was over double that of patients without delirium. Perhaps even more striking, is that we found a significant dose-response relationship between duration of delirium exposure and odds of worse clinical outcomes. For example, as compared to no delirium, three days of delirium was independently predictive of nearly four-fold greater 6-month mortality and nearly three-fold worse 6-month disability even after adjusting for relevant covariates. To our knowledge, this is the first prospective cohort study of hospitalized patients in an LMIC to assess the relationships between delirium and 6-month mortality and disability, providing new and robust data regarding comprehensive long-term functional outcomes of critical illness in a resource-limited setting through novel use of the WHODAS 2.0.

It is interesting to compare these data from our LMIC investigation with existing literature from high-income countries showing that delirium is independently predictive of worse long-term outcomes [2–12]. In 2004, it was shown that delirium exposure in mechanically ventilated ICU patients predicts a greater than three-fold increase in 6-month mortality [2], a value comparable to the results of this investigation (odds ratio 3.92 for maximum delirium duration). In keeping with our findings of a dose-response between delirium duration and both mortality and disability, others have found that each additional day of delirium in the ICU increases the hazard of 1-year mortality by 10%, [2, 3] and that a longer duration of delirium is independently associated with long-term cognitive and functional impairment [6–10]. Among hospitalized patients in the United States with acquired immunodeficiency syndrome, delirium has been associated with higher in-hospital mortality and greater need for long-term care [37, 38].

The mean age of this Zambian cohort is generally lower than most cohorts of patients included in US and European studies of delirium, which has important public health implications with regard to the high rates of death and disability seen in this younger, working-age patient population. There are several reasons why this may be the case. Zambia has a younger overall population and lower life expectancy compared to those in developed countries. Furthermore, this is a medically and socioeconomically vulnerable population with high rates of poverty, HIV, TB, malnutrition, and other comorbidities which, combined with inequitable access to healthcare, likely contributed to an overall younger and sicker hospital patient population. Finally, this patient population consists of general medical and surgical ward patients, who are generally younger than the ICU cohorts typically studied for delirium in developed countries.

This investigation providing outcomes at 6 months from Zambia represents a deviation from most previous literature generated in LMICs, which commonly report only in-hospital outcomes. A recent review [19] on critical care outcomes in resource-limited settings found only one study of post-intensive care outcomes [20]. No reports have documented long-term functional outcomes for survivors of critical illness in LMICs, many of whom are managed in non-ICU settings [19]. Our study is a response to the driving unmet need to expand our understanding of long-term outcomes from critical illness in LMICs [19, 39]. Our greater than 98% follow-up rate using mobile phones demonstrates that long-term follow-up of functional outcomes using the WHO Disability Assessment Schedule 2.0 is feasible in a resource-limited setting among patients with a high severity of illness and significant burden of delirium.

The high prevalence of delirium and severity of illness on hospital presentation warrants further study, particularly with regard to health system factors. Primary care in Zambia is undergoing a rapid transformation with an aim toward implementing the Sustainable Development Goals and advancing universal health coverage. However, the health system faces challenges associated with inequitable access and affordability of healthcare for those living in poverty, as well as insufficient human resources, frequent drug stockouts, and shortages of medical equipment, technology, and transportation [40]. Further exploration of whether structural issues in the healthcare system, including gaps in primary care, led to the high severity of illness and significant burden of delirium on hospitalization would be an objective of a future study. It should also be noted that this investigation was conducted at the country’s national referral hospital, so the population was likely skewed towards patients with more severe illness as those with less severe illness are usually managed at lower levels of the health system.

At the time of this investigation delirium was not routinely assessed in the hospital in which this study was conducted nor, to our knowledge, in other hospitals in the region. However, the University of Zambia Teaching Hospital has recently started a neurology residency training program, the first in the country, which is producing clinical neurologists who are trained to manage neurological conditions such as delirium.

This study has several other strengths. The sample size was large for a prospective cohort study of hospitalized patients (N = 711) and included both medical and surgical admissions. Duration of delirium exposure was evaluated using a validated tool for delirium assessment. All outcome assessment of participants was blinded to their delirium exposure. Disability was evaluated using a validated WHO tool for comprehensive functional assessment across multiple disability domains. Furthermore, seven *a priori* defined covariates including age, sex, education, income, severity of illness (UVA), HIV status, and antituberculosis treatment were controlled for in all regression analyses.

### Limitations

Several limitations must be discussed. First, a convenience sample approach was taken for participant enrollment because of the high volume of patients being admitted each day. We addressed this with a point prevalence study in the same setting immediately after completing enrollment of our primary cohort, in which we assessed all 330 patients admitted throughout a seven-day period. A similar delirium prevalence of 43.6% was found (vs. 47% in our primary cohort at enrollment), suggesting the convenience sample was likely representative of all admissions to the hospital. Second, due to staffing limitations, we only evaluated patients for delirium over the initial 3 days of hospitalization, which limited our ability to determine the predictive relationship between further delirium exposure and outcomes. Third, although we report 6-month mortality, reliance on verbal recall from next-of-kin limited our assurance regarding the exact date of death for those who died remotely between 1 and 6 months. Lastly, while the UVA SOI score itself was not predictive of mortality, we also ran models that controlled for MEWS score, which was predictive of 6-month mortality and disability, and qSOFA score and found that delirium remained independently predictive of the same clinical outcomes in a dose-dependent fashion.

## Conclusions

Delirium prevalence was high among hospitalized patients in this Zambian cohort with a significant burden of HIV and TB, and delirium duration was a strong independent predictor of 6-month mortality and disability. These data speak to the fact that delirium is important in predicting outcomes in hospitalized patients admitted to medical and surgical wards in sub-Saharan Africa, a point not previously known. Since delirium is not routinely included in daily patient monitoring in sub-Saharan African countries, there is a driving unmet need to understand better its epidemiology as a prognostically important form of acute organ dysfunction in resource-limited settings. We must build on these data to understand whether detection, prevention, and appropriate management of delirium (most of which consists of non-pharmacological interventions) can improve patient outcomes. Our results also demonstrate that long-term follow up of functional outcomes and the use of WHODAS 2.0 in patient populations with a high burden of critical illness is feasible in resource-limited settings, which has important implications for the design of future outcome studies of critical illness in LMICs.

## Supporting information

S1 Method Modified bCAM. (DOCX)
