# Supplementary material for: Delirium as a predictor of mortality and disability among hospitalized patients in Zambia
Source: PLoS One. 2021 Feb 11;16(2):e0246330. doi: 10.1371/journal.pone.0246330 (PMC7877643; doi:10.1371/journal.pone.0246330)
Supplement: S1 Method — (DOCX) [file pone.0246330.s001.docx]

| **MODIFIED BRIEF CONFUSION ASSESSMENT METHOD (bCAM)** | | | | |
| --- | --- | --- | --- | --- |
| **Feature 1: Acute Onset or Fluctuating Course** | | | | |
| **1A: Is the patient different than his/her baseline mental status?**  Directions: Ask the surrogate, "*Has the patient been more confused to you lately?*” | **No** | | | **Yes** |
| **1B: Has the patient had any fluctuation in mental status in the past 24 hours?**  Directions: Ask the surrogate, “*Have you noticed any fluctuations in the patient’s mental status where he/she appears to be more confused at some moments and less confused at other moments throughout the course of the day?*” | **No** | | | **Yes** |
| Positive if you answer **“yes”** to either **1A** or **1B**. | **Negative** | | | **Positive** |
| 🡺 *If “Positive”, proceed to Feature 2. If “Negative”, stop here, participant does not have delirium.* | | | | |
| **Feature 2: Inattention** | | | | |
| **2A: Numbers test for inattention**  Directions: Say to the patient, *“I am going to read you a series of 10 numbers. Whenever you hear the number ‘7’, indicate by squeezing my hand and then letting go.”* Read numbers from the following number list in a normal tone.  **3** **7** **5 8 7 6 7 7 4 2**  Scoring: Errors are counted when the patient fails to squeeze on the number ‘7’ and when the patient squeezes on any number other than ‘7’. | **Score (out of 10): _____** | | | |
| **2B: Months backwards test for inattention**  Directions: Say to the patient, *“Can you name the months backwards starting from December to July”*  Scoring: The score is the number of months the patient correctly got in order. If the patient prematurely stops, then the missed months are considered errors. If the patient skips a month, each month skipped is considered an error. | **Score (out of 6): _____**  If score 5 or 6, did the patient take longer (>20 seconds) than usual to complete this task?  No Yes | | | |
| Positive if **2A** is less than **9** or **2B** is less than **5**. | **Negative** | | **Positive** | |
| 🡺 *If “Positive”, proceed to Feature 3. If “Negative”, stop here, participant does not have delirium.* | | | | |
| **Feature 3: Altered Level of Consciousness** | | | | |
| **3: Richmond Agitation and Sedation Score (RASS)**    What is the patient’s actual RASS? In patients with a RASS of -4 or -5, the B-CAM is not assessable.   \| Score \| Label \| Description \| \| --- \| --- \| --- \| \| +4 \| Combative \| Combative, violent, immediate danger to staff \| \| +3 \| Very Agitated \| Pulls to remove tubes or catheters, aggressive \| \| +2 \| Agitated \| Frequent non-purposeful movement, fights ventilator \| \| +1 \| Restless \| Anxious, apprehensive, movements not aggressive \| \| 0 \| Alert & Calm \| Spontaneously pays attention to caregiver \| \| -1 \| Drowsy \| Not fully alert, but has sustained awakening to voice (eye opening and contact greater than 10 seconds) \| \| -2 \| Light Sedation \| Briefly awakens to voice (eyes open and contact less than 10 seconds) \| \| -3 \| Moderate Sedation \| Movement or eye opening to voice (no eye contact) \| \| -4 \| Deep Sedation \| No response to voice, but movement or eye opening to physical stimulation \| \| -5 \| Unarousable \| No response to voice or physical stimulation \| | **Score (-3 to +4): _____** | | | |
| Positive if the Actual RASS score is anything other than **“0”**. | **Negative** | **Positive** | | |
| 🡺 *If “Positive”, stop here, participant has delirium. If “Negative”, proceed to Feature 4.* | | | | |
| **Feature 4: Disorganized Thinking** | | | | |
| **4A: Yes/No Questions**  (Use either Set A or Set B, alternate on consecutive days if necessary):  **Set A Set B**  1. Will a stone float on water? 1. Will a leaf float on water?  2. Are there birds in trees? 2. Are there elephants in trees?  3. Does one bag of sugar weigh more 3. Do two bags of sugar weigh more than  than two bags of sugar? one bag of sugar?  4. Can you use a hammer to pound 4. Can you use a hammer to cut  a nail? wood?  **Score _______** (Patient earns 1 point for each correct answer out of 4).  **4B: Command**  Say to patient: “*Hold up this many fingers*” (Examiner holds two fingers in front of patient). “*Now do the same thing with the other hand*” (Not repeating the number of fingers). (If patient is unable to move both arms, for the second part of the command ask patient “*Add one more finger*”).  **Score ________** (Patient earns 1 point if able to successfully complete the entire command) | **Combined Score (4A+4B)**  **_______ (out of 5)** | | | |
| Positive if the combined score is less than **5**. | **Negative** | **Positive** | | |
| 🡺 *If “Positive”, participant has delirium. If “Negative”, participant does not have delirium.* | | | | |
| **Overall B-CAM** (Features 1 and 2 and either Feature 3 or 4) | **Negative** | **Positive** | | |
|  | **Unassessable**  (Unresponsive to voice) | | | |
